# Normal Thoracic Radiographic Appearance of the Cynomolgus Monkey (*Macaca fascicularis*)

**DOI:** 10.1371/journal.pone.0084599

**Published:** 2014-01-08

**Authors:** Liang Xie, Qinming Zhou, Shigang Liu, Qingyuan Wu, Yongjia Ji, Lujun Zhang, Fan Xu, Wei Gong, Narayan D. Melgiri, Peng Xie

**Affiliations:** 1 Department of Neurology, The First Affiliated Hospital at Chongqing Medical University, Chongqing, China; 2 Chongqing Key Laboratory of Neurobiology, Chongqing, China; 3 Institute of Neuroscience, Chongqing Medical University, Chongqing, China; 4 Suzhou Xishan Zhongke Laboratory Animal Co., Ltd., Suzhou, China; Wayne State University, United States of America

## Abstract

**Background:**

The cynomolgus monkey (*Macaca fascicularis*) has been increasingly used as a non-human primate model in biomedical research. As establishing baseline thoracic radiography for the cynomolgus monkey is essential, we tested the hypothesis that age and sex may affect the thoracic radiography parameters of this species.

**Methods:**

Here, 697 healthy cynomolgus monkeys were segregated by sex and age (three age groups: 25–36 months, 37–48 months, 49–60 months). The lung length (LL), maximal interior thoracic depth (TD), maximal interior thoracic breadth (TBr), cardiac silhouette breadth (CBr), cardiothoracic ratio (CR), right and left costophrenic angles (RCA and LCA), and right hilar height ratio (R-HHR) were assessed by chest film. Statistical analysis was applied to examine the effect of age, sex, and age × sex interactions.

**Results:**

Significant effects by age were shown for LL, TD, TBr, CBr, and CR. Significant effects by sex were found for TD, TBr, CBr, CR, and R-HHR. Significant effects by age × sex were observed for TD, TBr, CBr, and CR. Both TD and TBr increased with age in both sexes, and both were significantly higher in males than in females in the group aged 49–60 months. CBr increased with age and was significantly higher in males than in females across all age groups. CR declined with age and was significantly higher in males than females across all age groups, and CR was similar or slightly higher relative to those previously found in other non-human primate species. As to the other parameters with no significant sex nor age-related differences, the R-HHR was greater than 1.00, and the angulation of bilateral costophrenic angles were sharp.

**Conclusions:**

The thoracic radiographic parameters for the healthy cynomolgus monkey presented here should prove useful in veterinary practice, research involving non-human primate models of respiratory or cardiovascular disorders, and morphological studies on cynomolgus monkeys.

## Introduction

The cynomolgus monkey (*Macaca fascicularis*), an Old World monkey, has been increasingly used as a non-human primate model in biomedical research, as the species is more anatomically and physiologically homologous to humans as compared with other animal models (e.g. rats, pigs), and also shares behavioral characteristics with humans.

Two-dimensional projectional X-ray (“plain film”) thoracic radiography is a well-established, routine, non-invasive technique used in colony management and experimental protocols at many non-human primate facilities. Thoracic radiography of non-human primates is widely applied in three respects. First, thoracic radiography is used as a diagnostic modality in veterinary clinical medicine of non-human primates [1]. Second, thoracic radiography has been widely applied to animal disease model research involving non-human primates [2], [3]. Third, radiography is also useful for primatologists and other researchers interested in morphometric studies of primate anatomy.

Normal baseline measures of thoracic radiographic parameters in non-human primates are essential for defining abnormalities. Several studies on the normal radiographic anatomy of rhesus macaques [4], [5], marmosets [6], formosan monkeys [7], cynomolgus monkeys [8], and pet macaques from Sulawesi (*Macaca nigra* and *Macaca tonkeana*) [9], [10] have already been conducted.

Although there has been one published study which has reported the normal cardiothoracic ratio of the feral cynomolgus monkey [8], other valuable thoracic radiographic indices of the cynomolgus monkey have not been previously reported (e.g., lung length, hilar height ratio). Furthermore, the sample size of the above study was relatively small. In addition, most cynomolgus monkeys used in scientific research are reared and observed in captivity, and thoracic radiography of captive cynomolgus monkeys has not been a focus of previous investigation.

In order to build an integrated, high-quality foundation for future cynomolgus monkey-based research, one of the first and most important investigations should be to measure and establish comprehensive and accurate indices of thoracic radiography for this species. Therefore, the aim of this study is to provide a description and establish comprehensive and accurate reference thoracic radiographic indices based on 697 cynomolgus monkeys aged 25–60 months. We also hypothesized that age and sex may affect several thoracic radiography parameters.

## Methods

### Subjects and Ethical Statement

This study was conducted based on raw thoracic radiographic data from 697 healthy cynomolgus monkeys (391 males and 306 females; age range: 25–60 months) (Table 1), which were collected during biannual routine health check-ups. All subjects were housed at the Xishan Zhongke Laboratory Animals Co. Ltd. (Suzhou, China), an Association for the Assessment and Accreditation of Laboratory Animal Care International (AAALAC)-accredited company.

**Table 1:** Sample Sizes by Age and Sex.

Age (months)	Males andFemales (n)	Males (n)	Females (n)
25–36	356	184	172
37–48	253	126	127
49–60	88	81	7
Total	697	391	306

All subjects were housed in either same-sex social groups or mixed-sex social groups that were formed when the monkeys were six months of age and were stable thereafter. The groups were housed in 8×3×3 m indoor pens that were maintained within the temperature range of 18–29°C with a relative humidity ranging from 40–70% (varying with natural seasonality). The subjects were given water *ad libitum* and fed twice daily with fresh fruits, vegetables, and compound high-nutrition monkey food. The living environment and animal care procedures have been previously reported [11]. These monkeys were determined to be healthy by history and veterinary examination and were free of tuberculosis and Herpes B virus. All female subjects were non-pregnant.

The research complied with protocols approved by the Animal Care and Use Committee of Chongqing Medical University (Approval No: 20100031), adhered to the legal and regulatory requirements of the People’s Republic of China (P.R.C.), and the American Society of Primatologists (ASP) Principles for the Ethical Treatment of Non-Human Primates. No animals were sacrificed in this study.

### Radiographic Examination

The protocol for thoracic radiography has been described in our previous study [5]. The digital detector was exposed to X-rays at 60 KVp with an approximate detector-to-tube distance of 1 m. Exposure times were no greater than 0.1 s, resulting in 4.0 mAs of exposure. After ketamine anesthesia (10 mg/kg, IM), animals were positioned on a platform under the detector with arms extended laterally by two assistants wearing appropriate shielding to avoid radiation exposure. Images were obtained during the inspiratory phase of respiration. Right-to-left lateral and posteroanterior thoracic radiographs were obtained from all subjects.

### Radiographic Measurement

**Figure 1:**
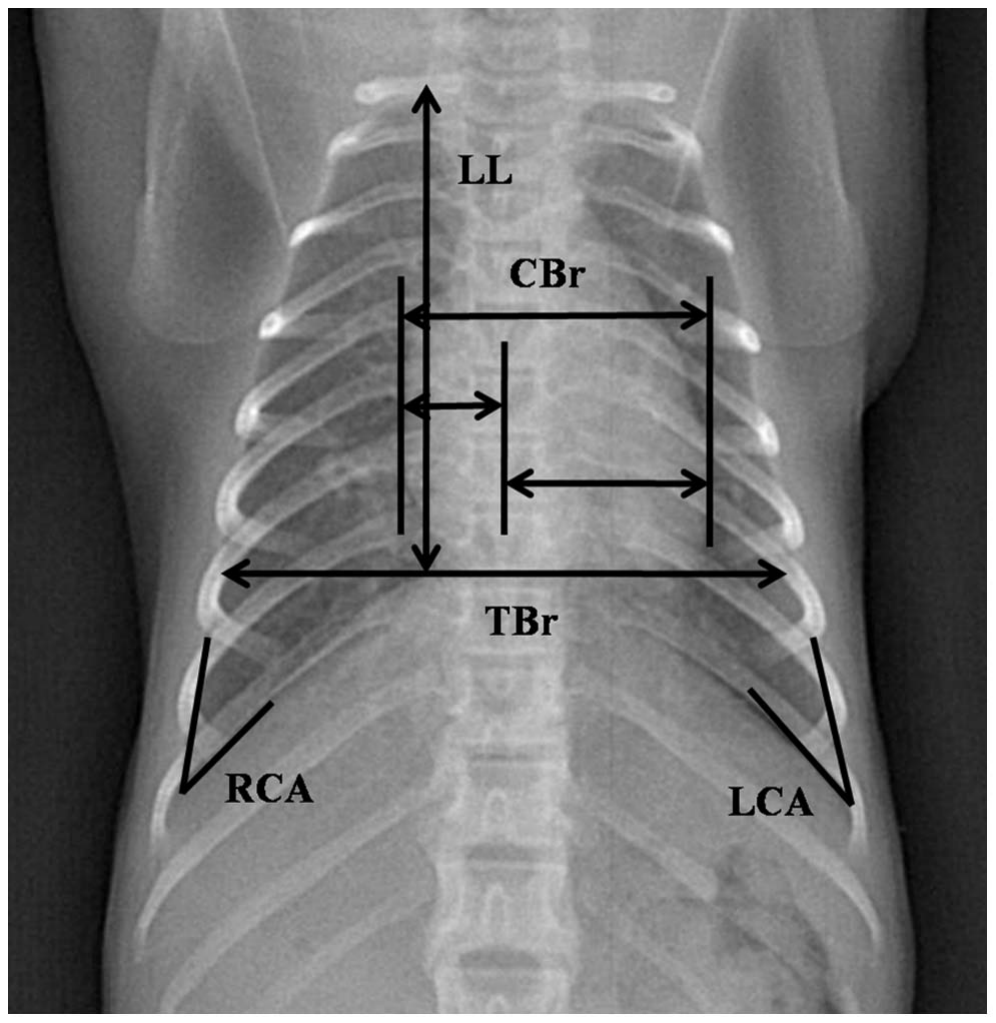
Normal Posteroanterior Thoracic Radiograph of the Cynomolgus Monkey. The lung length (LL), maximal interior thoracic breadth (TBr), maximal breadth of cardiac silhouette (CBr), right and left costophrenic angles (RCA and LCA), and cardiothoracic ratio (CR = CBr/TBr) are indicated.

**Figure 2:**
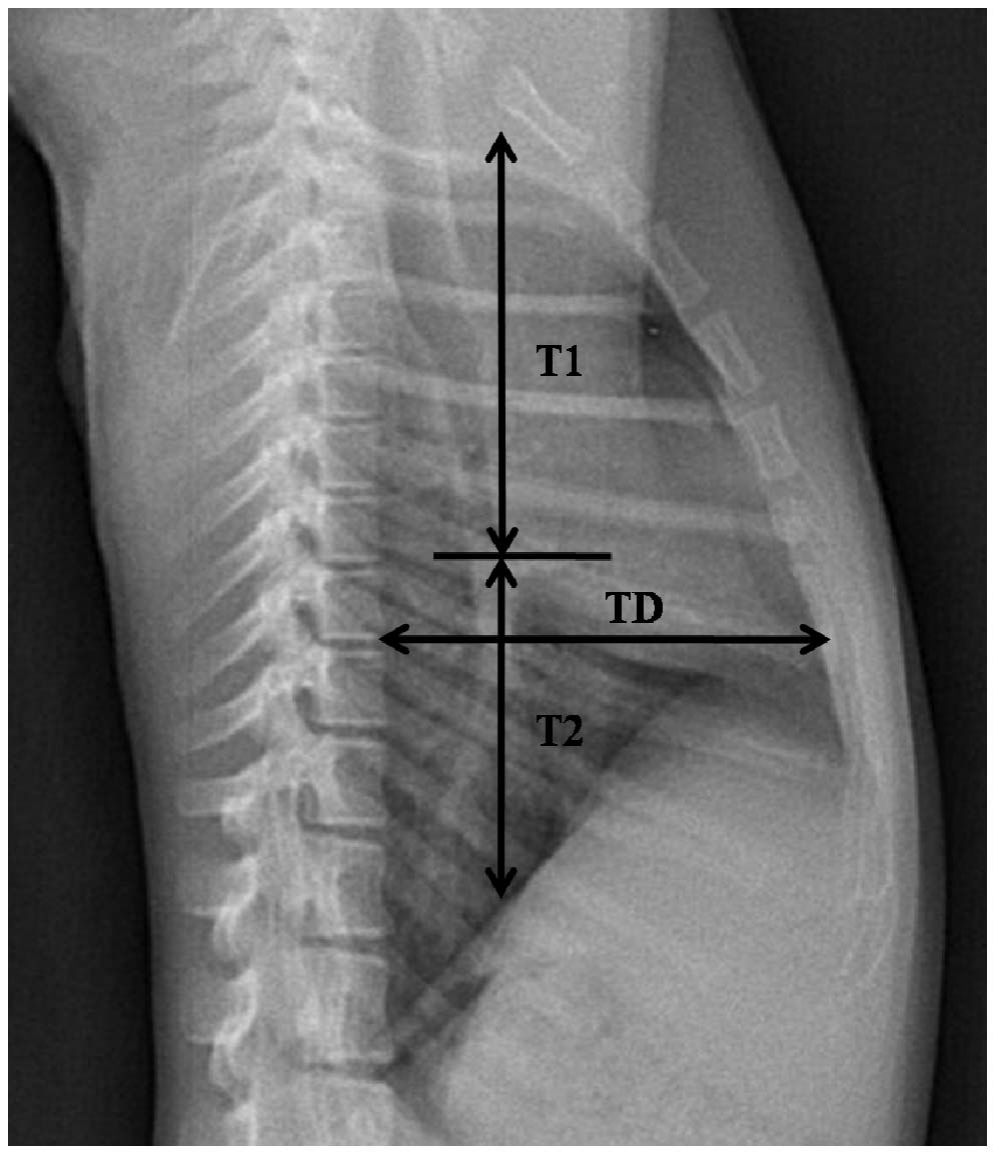
Normal Right-to-left Lateral Thoracic Radiograph of the Cynomolgus Monkey. The maximal interior thoracic depth (TD) and right hilar height ratio (R-HHR) are indicated. R-HHR was calculated as the distance from the pulmonary apex to the hilus pulmonis (T1) divided by the distance from the hilus pulmonis to the diaphragm (T2).

The digital radiographs were interpreted and measured by a licensed, blinded veterinarian. Thoracic measurements (Fig. 1–2) were performed with DXRay Diagnost software which is included in the digital radiography system (PLX8200, Nanjing Perlove Medical Equipment Co., Ltd, Nanjing, China), the software allowed us to view the digital radiographs on the computer and measure the radiographs by built-in tools. After taking and conserving the films, we can measure them accurately by the software. The lung length (LL) was defined as the distance from the tubercle of the first rib to the top of the dome of the right diaphragm [12]. The maximal interior thoracic depth (TD), obtained from lateral films, was defined as the distance between the ventral surface of the vertebral body and the posterior sternum at the level of the top of the right diaphragm. The maximal interior thoracic breadth (TBr) was defined as the linear distance between the internal margins of the ribs near the superior margin of the hemidiaphragm [9]. The cardiac silhouette breadth (CBr) was defined as the sum of the distance from the midline to the right border of the cardiac silhouette plus the distance from the midline to the left border of the cardiac silhouette [9]. The cardiothoracic ratio (CR) was defined as CBr divided by TBr [9]. The bilateral costophrenic angles (CA), consisting of the right costophrenic angle (RCA) and the left costophrenic angle (LCA), was defined from the right and left sides where the diaphragm meets the ribs [13]. The right hilar height ratio (R-HHR) was obtained from lateral radiography by drawing a line parallel to the thoracic spine from the highest point of the pulmonary apex to the diaphragm; this did not always end at the highest point on the diaphragm. An intersecting line then was drawn from the midpoint of the hilus pulmonis perpendicular to the first line. R-HHR was defined as the ratio of the distance from the pulmonary apex to the hilus pulmonis (T1) to the distance from the hilus pulmonis to the diaphragm (T2) [14].

### Statistical Analysis

All data were presented as means ± standard deviations (Tables 2–4), and all data processing, data management, and statistical analysis were performed using SPSS version 18 software (SPSS, Chicago, IL).

**Table 2:** Measurements of Cynomolgus Monkeys Aged 25–36 Months*.

Parameters (unit)	Males and Females (n = 356)	Males (n = 184)	Females (n = 172)	Range
LL (mm)	64.19±6.97	62.44±6.52	66.07±6.98	47.03–85.59
TD (mm)	60.69±4.06	60.96±4.25	60.40±3.83	48.68–76.59
TBr (mm)	74.89±4.81	75.47±4.46	74.26±5.09	63.94–96.44
CBr (mm)	43.67±3.00	44.34±2.82	42.95±3.02	33.93–52.53
CR	0.58±0.04	0.59±0.04	0.58±0.04	0.42–0.64
T1 (mm)	48.69±4.10	48.86±4.29	48.51±3.90	38.35–67.66
T2 (mm)	39.08±4.07	39.02±4.28	39.13±3.85	28.70–50.16
R-HHR	1.25±0.12	1.26±0.13	1.25±0.12	1.01–1.84
RCA (°)	34.13±4.53	34.55±4.84	33.68±4.15	24.14–47.76
LCA (°)	35.45±4.46	36.00±4.66	34.86±4.16	21.59–49.85

Range is defined as the lowest observed value to the highest observed value. CR = CBr/TBr. R-HHR = T1/T2.

**Table 3:** Measurements of Cynomolgus Monkeys Aged 37–48 Months*.

Parameters (unit)	Males and Females (n = 253)	Males (n = 126)	Females (n = 127)	Range
LL (mm)	68.65±7.10	67.16±6.71	70.11±7.20	49.67–96.42
TD (mm)	63.51±5.34	63.96±5.25	63.06±5.40	49.38–76.41
TBr (mm)	79.56±5.12	79.62±5.39	79.51±4.85	67.65–97.29
CBr (mm)	45.50±3.49	46.42±3.34	44.59±3.42	33.01–55.62
CR	0.57±0.04	0.58±0.04	0.56±0.04	0.43–0.64
T1 (mm)	51.98±4.06	52.15±4.41	51.82±3.70	41.85–63.76
T2 (mm)	42.07±4.91	41.81±5.28	42.33±4.51	29.53–62.73
R-HHR	1.25±0.13	1.26±0.14	1.23±0.11	1.00–1.60
RCA (°)	33.76±4.76	34.38±5.14	33.16±4.28	23.62–53.74
LCA (°)	34.62±4.81	34.42±4.93	34.81±4.70	25.59–50.44

Range is defined as the lowest observed value to the highest observed value. CR = CBr/TBr. R-HHR = T1/T2.

**Table 4:** Measurements of Cynomolgus Monkeys Aged 49–60 Months*.

Parameters (unit)	Males and Females (n = 88)	Males (n = 81)	Females (n = 7)	Range
LL (mm)	82.15±8.94	82.41±9.14	79.13±5.70	61.38–107.55
TD (mm)	75.92±5.51	76.75±4.79	66.24±3.94	59.31–85.75
TBr (mm)	95.21±7.01	95.77±6.91	88.80±4.74	81.05–117.81
CBr (mm)	53.02±4.65	53.7±04.13	45.17±2.91	41.95–63.49
CR	0.56±0.04	0.56±0.04	0.51±0.03	0.47–0.64
T1 (mm)	57.06±4.72	57.54±4.54	51.53±2.99	47.29–69.58
T2 (mm)	46.88±5.14	47.09±5.25	44.49±2.95	31.94–59.10
R-HHR	1.23±0.11	1.23±0.11	1.16±0.07	1.03–1.56
RCA (°)	32.50±4.09	32.48±4.05	32.68±4.87	24.05–43.03
LCA (°)	33.19±3.66	33.10±3.76	34.32±2.17	23.66–41.90

Range is defined as the lowest observed value to the highest observed value. CR = CBr/TBr. R-HHR = T1/T2.

First, a two-way analysis of variance (ANOVA) was used to examine the effect of age, sex, and age × sex interactions (Table 5). Moreover, the post-hoc test by one-way ANOVA was executed to distinguish differences in the three age groups within the same sex. Finally, a Student’s *t*-test was applied to detect significant differences between male and female subjects in the same age group. The significance level was set to 0.05.

**Table 5:** Summary of Age and Sex Effects on Thoracic Measurements.

Parameters (unit)	Age	Sex	Interaction
LL (mm)	F(2, 691) = 80.46, P<0.01	F(1, 691) = 1.18, NS	F(2, 691) = 2.83, NS
TD (mm)	F(2, 691) = 80.92, P<0.01	F(1, 691) = 36.61, P<0.01	F(2, 691) = 13.90, P<0.01
TBr (mm)	F(2, 691) = 170.04, P<0.01	F(1, 691) = 13.93, P<0.01	F(2, 691) = 5.28, P<0.01
CBr (mm)	F(2, 691) = 53.73, P<0.01	F(1, 691) = 71.77, P<0.01	F(2, 691) = 14.49, P<0.01
CR	F(2, 691) = 22.21, P<0.01	F(1, 691) = 25.72, P<0.01	F(2, 691) = 5.35, P<0.01
R-HHR	F(2, 691) = 2.78, NS	F(1, 691) = 4.42, P<0.05	F(2, 691) = 0.74, NS
RCA (°)	F(2, 691) = 1.59, NS	F(1, 691) = 0.95, NS	F(2, 691) = 0.33, NS
LCA (°)	F(2, 691) = 3.61, NS	F(1, 691) = 0.07, NS	F(2, 691) = 2.62, NS

NS, not significant. CR = CBr/TBr. R-HHR = T1/T2.

Second, the Spearman rank-correlation test was employed to determine whether the cardiothoracic ratio (CR) and cardiac silhouette breadth (CBr) correlated significantly with body weight. The CR did not significantly correlate with the body weight of either males (r = 0.021, p = 0.683, n = 391) or females (r = 0.055, p = 0.337, n = 306). However, the CBr did significantly correlate with body weight of males (r = 0.611, p<0.01, n = 391) and females (r = 0.321, p<0.01, n = 306).

## Results

**Figure 3:**
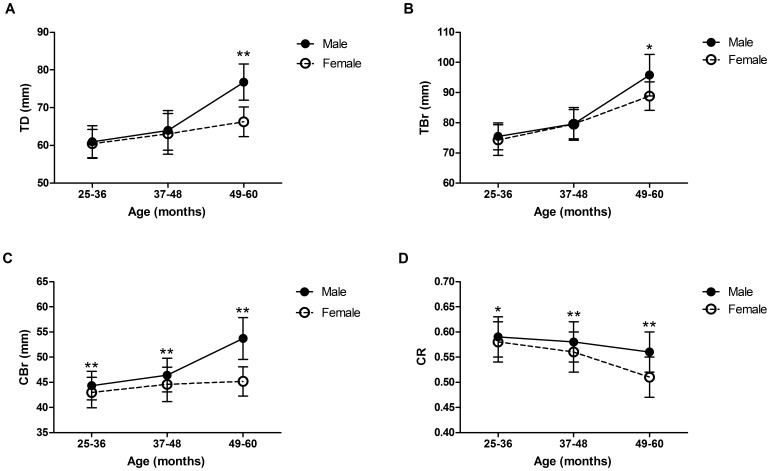
Changes to TD, TBr, CBr, and CR (CBr/TBr) in Males and Females with Age. Data are presented as means ± SDs at each point. Significant differences between the sexes in the respective age groups were assessed by Student’s *t*-test (*p<0.05, **p<0.01).

Reference values and ranges of thoracic radiography measurements by age and sex are reported in Tables 2–4. The results from the ANOVA tests examining the effects of age, sex, and age × sex interactions on the thoracic radiographic values are presented in Table 5. Significant effects by age were shown for LL, TD, TBr, CBr, and CR. Significant effects by sex were found for TD, TBr, CBr, CR, and R-HHR. Significant effects by age × sex were observed for TD, TBr, CBr, and CR. Both TD and TBr increased with age in both sexes, and both were significantly higher in males than in females in the group aged 49–60 months (Fig. 3A, 3B). CBr increased with age and was significantly higher in males than in females across all age groups (Fig. 3C). CR declined with age and was significantly higher in males than females across all age groups (Fig. 3D).

## Discussion

In this study, chest films from 697 healthy cynomolgus monkeys were analyzed in order to describe the normal thoracic radiographic appearance of this macaque species by age and sex and to determine the effects of age and sex on thoracic radiographic parameters. These indices are helpful in diagnosing respiratory and cardiovascular disorders and characterizing these disease processes in animal disease models [15]. Though more advanced imaging techniques such as computed tomography (CT) and magnetic resonance imaging (MRI) have emerged, projectional X-ray (“plain film”) radiography remains the most rapid, cost-effective imaging modality for non-human primates used at monkey bases and laboratories globally. As no study has systematically measured chest film parameters on cynomolgus macaques, it is essential to construct reference indices of thoracic-based parameters for this species.

Thoracic radiography can be applied as a tool for evaluating developmental morphology and thoracic volume; specifically, LL, TD, and TBr can be used as evaluation parameters in radiographic film analysis [16]. Abnormal indices likely indicate thoracic dysplasia, which can lead to more serious respiratory disorders. Here, LL, TD, and TBr all increased with age; interestingly, similar age-related differences in thoracic parameters have also been reported in rhesus macaques [5]. The phenomenon is obviously related to pubertal skeletal growth. On account of the higher sexual dimorphic growth rate in male relative to female cynomolgus macaques with advancing age, LL, TD, and TBr were significantly higher in males than in females in the 49–60 month age group [17], [18].

In human studies, the cardiothoracic ratio (CR) is a well-established measurement of cardiac size and provides prognostic information on congenital cardiac defects and acquired heart disease [19], [20]. Non-human primates display similar heart diseases to humans [1], [7], and there have been five studies reporting CRs in non-human primates [4], [5], [7], [8], [9]. With regard to these studies, the mean CR of captive cynomolgus monkey found here was most similar to that of the *Macaca tonkeana* (mean CR = 0.599) [9] and the rhesus monkey (*Macaca mulatta*) [5] (mean CR = 0.59), but slightly higher than that of the *Macaca cyclopis* (mean CR = 0.528) [7], *Macaca nigra* (mean CR = 0.522) [9], and feral cynomolgus monkeys from Indonesia (mean CR = 0.550) [8]. There are two hypotheses regarding these differences. First, inter-species variability or differences in origin between the same species may have contributed to these differences. Second, the differences in age structure among the study groups may have lead to these differences. Moreover, in contrast to the aforementioned studies [5], [7], [8], [9], our study found that CRs declined with age. This pattern of age-related changes suggests that the cardiac growth rate is slower than the TBr. In addition, the relationship between CR and CBr (CR x TBr) and body weight was analyzed. The CR did not significantly correlate with the body weight of either males (r = 0.021, p = 0.683) or females (r = 0.055, p = 0.337). Similarly, Schillaci et al. has reported no significant relationship between CR and body weight in adult feral cynomolgus monkeys and pet macaques from Indonesia [8], [9]. In contrast, the CBr did significantly correlate with the body weight of males (r = 0.611, p<0.01, n = 391) and females (r = 0.321, p<0.01, n = 306). We speculate that the significant CBr-body weight correlation (and the lack of a significant CR-body weight correlation) is related to pubertal skeletal growth, as this phenomenon would affect CBr but not CR.

The hilar height ratio (HHR) is used to evaluate the position of the hilus in the hemithorax. Thus, an abnormal HHR aids in diagnosing alterations in pulmonary volume such as lobar collapse and over-aerations [14]. This study is the first to report the right HHR (R-HHR) of the healthy cynomolgus monkey. We found that the R-HHR values were relatively constant across all three age groups (range: 1.00–1.84), which is consistent with human studies reporting normal R-HHR values exceeding 1.00 [14]. Compared with our previous findings in rhesus monkeys [5], R-HHR values were slightly lower in cynomolgus monkeys as compared to rhesus monkeys on an age group basis, which may be attributed to inter-species variation. We also observed that R-HHR values were higher in males than in females, but no explanation is available for this sex-based variation.

The presentation of costophrenic angles in chest films are effective diagnostic indicators of pleural effusions [21]. The angulation of bilateral costophrenic angles are typically sharp under normal conditions and typically blunted in diseased states. Here, the bilateral costophrenic angles were clear and sharp and varied in size from 22 to 54 degrees, which is similar to previous observations in rhesus monkeys [4] and capuchin monkeys [13]. There were no sex- nor age-associated differences observed with respect to bilateral costophrenic angles.

## Limitations

There are two notable limitations in our study. First, the sample size of female monkeys in the 49–60 months age group was limited to only seven subjects. Second, all subjects enrolled in our study were under 60 months; thus, the normal appearance of chest films for cynomolgus monkeys older than 60 months should be assessed in future studies with the goal of constructing a more complete database of thoracic radiographs while determining the effects of age and sex across a broader range of age groups.

## Conclusion

The thoracic radiographic parameters for the healthy cynomolgus monkey presented here should prove useful in veterinary practice, research involving non-human primate models of respiratory or cardiovascular disorders, and morphologic studies on cynomolgus monkeys. As the CR is a commonly-used diagnostic index in veterinary medicine, we recommend that the age and sex of the subject should be carefully considered because age and sex can affect CR.
