# Correction: Normal Thoracic Radiographic Appearance of the Cynomolgus Monkey (*Macaca fascicularis*)

**DOI:** 10.1371/journal.pone.0093028

**Published:** 2014-03-14

**Authors:** The PLOS ONE Staff

There is an error in the affiliation for the authors. The correct author affiliation is “the First Affiliated Hospital of Chongqing Medical University.”
